# Lebanese Medicinal Plants with Ophthalmic Properties

**DOI:** 10.3390/ph18020155

**Published:** 2025-01-24

**Authors:** Jeanne Andary, Haitham El Ballouz, Rony Abou-Khalil

**Affiliations:** 1 Faculty of Health Sciences, Modern University for Business and Science, Beirut P.O. Box 113-7501, Lebanon; 2 Department of Optics and Optometry, Faculty of Health Sciences, American University of Science and Technology, Beirut P.O. Box 16-6452, Lebanon; helballouz@aust.edu.lb; 3 Biology Department, Faculty of Arts and Sciences, Holy Spirit University of Kaslik, Jounieh P.O. Box 446, Lebanon; ronyaboukhalil@usek.edu.lb

**Keywords:** herbal medicine, eye, Lebanon, ophthalmic treatment, ethnopharmacology

## Abstract

Lebanon benefits from a rich biodiversity, with medicinal and aromatic plants (MAPs) representing an important part of the country’s natural wealth; however, limited data are available documenting medicinal plants being employed in eye health. This review is the first to document Lebanese medicinal plants with ophthalmic characteristics and phytochemistry that might be beneficial in the development of new, accessible, and efficient ocular medications. In this study, we searched for studies on ocular therapeutic plants using known resources, including PubMed, ScienceDirect, and Google Scholar, and confirmed these plants’ presence within the Lebanese flora. The efficacy of 52 species from 28 families, including two endemic species (*Crepis libanotica* and *Salvia libanotica*), has been documented. Their Latin names, regional names, ocular medical applications, the plant parts used, and preparation forms are detailed below. The largest number of species belongs to the Lamiaceae family (21%), followed by Asteraceae (14%) and Solanaceae (7%). The most commonly used plant parts are the stems, leaves, and seeds. Ocular treatments fall into several categories: inflammation, infection, irritation, dry-eye, eyewash, the prevention or delay of cataracts, and general eye problems. A significant percentage (68%) of the medicinal plants target the anterior part of the eye. Some of the reported plants can be harmful to the eyes and should be handled with caution. The Lebanese medicinal plants listed, constituting a local heritage with global importance, could be used for treating ophthalmic ailments and require special screening and preservation.

## 1. Introduction

For centuries, herbal medicine has been used to treat a wide range of human disorders, with at least 80% of the world’s population, primarily in developing countries, relying on it for primary healthcare [1]. Since the beginning of civilization, humans have used resources from fauna and flora to treat eye diseases [2]. Pharmacologically active preparations that modulate eye activity have been utilized for over 2000 years, with Atropa belladonna extracts originally being used for pupillary dilatation [3]. Owing to their low cost, herbal medicines are increasingly preferred over contemporary pharmaceuticals, and conservative remedies are still popular for use in reversible ailments.

The Mediterranean Basin, with its mild climate and millennia-old population, is rich in plant diversity, including rare and endemic plants [4]. The daily Mediterranean diet includes vegetables, fruits, and spices, with both cultivated and wild food species providing essential nutritional value and medicinal properties [5]. Lebanon, located on the eastern shore of the Mediterranean Sea, benefits from a rich biodiversity [6], hosting more than 4500 plant species, 2863 of which are considered native, and an endemism rate of 12% [7]. A range of conventional herbal eye drops made from a variety of medicinal plants can be used to treat ocular diseases [2]. The development and exploration of a country’s untapped medical knowledge is necessary for the better management of ocular disorders. Therefore, documenting and categorizing the nation’s currently unknown medical information is crucial in improving the management of eye illnesses.

The purpose of this study was to highlight, for the first time, numerous Lebanese medicinal plants that have been traditionally used to treat eye disorders, which could be further researched for use in various ocular diseases. It is the right of the local population to better understand, use, and develop their indigenous resources. Additionally, this study can serve as a valuable starting point for researchers to develop new and more effective ocular formulations that respect plant biodiversity and environmental sustainability.

## 2. Major Ocular Diseases

**Figure 1:**
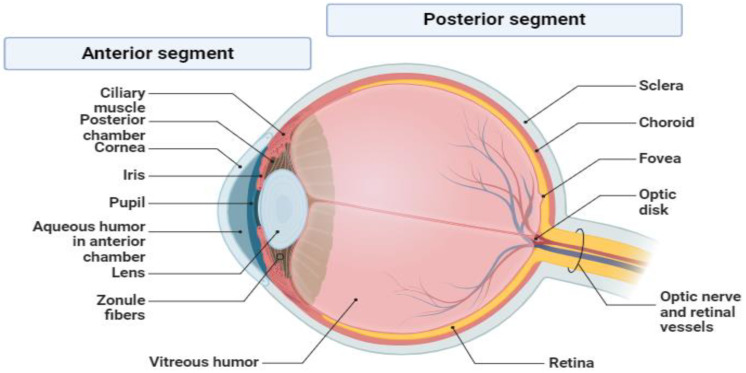
Anatomy of the eye with the anterior and posterior segments, created using Biorender.com [13].

The eye is a paired organ located in the orbital cavity, responsible for capturing images transmitted to the cortical vision center [8]. The anterior segment of the eye includes ocular structures from the anterior part of the cornea to the posterior part of the lens, as well as the trabecular meshwork (TM) and aqueous humor [9] (Figure 1). Posterior-segment eye disease (PSED) is commonly defined as comprising diseases of the retina, choroid, and optic nerve and primarily includes glaucoma, age-related macular degeneration (AMD), and diabetic retinopathy (DR) [10]. Ocular diseases affect vision and can lead to irreversible blindness and the loss of visual acuity. However, many causes of vision impairment can be prevented or treated [11].

At least 2.2 billion people worldwide suffer from visual impairment, with at least one billion having conditions that could have been avoided or remain untreated. The World Health Organization estimates that eye conditions are disproportionately more prevalent in low- and middle-income countries and medically underserved populations. It is also estimated that 11.9 million people globally suffer from moderate or severe vision impairment or blindness due to glaucoma, diabetic retinopathy, and trachoma conditions that could have been prevented [12].

### 2.1. Eye Infections

The eye is one of the most sensitive organs and is constantly exposed to various environmental agents. Tears contain several substances that help protect against infection, while the eyelids and eyelashes shield the ocular surface from the environment and help keep the surface of the eye moist. Occasionally, these defense mechanisms can be disrupted, leading to ocular problems [14]. Due to continuous exposure to the external environment, the eyes are susceptible to infections caused by bacteria, fungi, parasites, or viruses. The range of conditions and diseases that affect the eye vary widely, from redness to loss of vision. Most of these infections present clinically as infections of the anterior segment that include blepharitis, styes, conjunctivitis, corneal ulcers, and keratitis; infections of the lacrimal system, such as canaliculitis and dacryocystitis; or more severe infections affecting the orbit and the posterior segment, like orbital cellulitis and endophthalmitis [15].

### 2.2. Cataracts, Dry-Eye, and Allergies

Cataracts are the leading cause of visual impairment worldwide and are defined by the presence of lens opacities or the loss of transparency [16]. Cataracts resulting from long-term light exposure are caused by photo-oxidation that decomposes the biological components of the lens, primarily crystallin. The oxidized thiol groups in crystalline molecules form disulfide bonds, leading to crystalline aggregation and cataract formation [17].

The term ‘allergic conjunctivitis’ refers to a group of hypersensitivity disorders that affect the eyelid, conjunctiva, and/or cornea. The primary goal in managing ocular allergies is to identify the causes and prevent recurrence by eliminating them [18].

The Tear Film and Ocular Surface Society Dry Eye Workshop II (TFOS DEWS II) defines dry-eye disease as follows: In addition to causing cytotoxic effects and retinal ganglion cell loss, oxidative stress damages the trabecular meshwork, obstructing the outflow of aqueous humor and raising the intraocular pressure [19].

### 2.3. Glaucoma, Eye Cancer, and Diabetic Retinopathy

Glaucoma causes a progressive, irreversible loss in retinal ganglion cells, damage to the optic nerve, and loss of vision, both with and without increased intraocular pressure. However, intraocular pressure remains the most important modifiable risk factor. Oxidative stress has cytotoxic effects and causes, leading to retinal ganglion cell death and damage to the trabecular meshwork, which obstructs the outflow of aqueous humor and results in elevated intraocular pressure [20].

Diabetic retinopathy (DR) is the most common microvascular complication of diabetes mellitus (DM) and a leading cause of visual loss in working-age populations. Inflammation and retinal neurodegeneration may be involved in DR as independent pathogenesis pathways. The development of agents targeting molecules in these pathways may provide new therapeutic treatments for DR [21].

Eye cancer is a rare disease, with a lower occurrence compared to other forms of cancer, and it is generally less invasive. It can affect either the outer parts of the eye (extraocular cancer) or the eyeball itself (intraocular cancer). In adults, the most common intraocular cancers are melanoma and lymphoma, while in children, retinoblastoma is the most common and may be extraocular or intraocular [22].

## 3. Methodology

Data collection was carried out through an extensive review of the literature. We initially searched for studies on ocular therapeutic plants in well-known resources, using specific keywords such as medicinal plants, ocular diseases, and ophthalmic plants.

**Figure 2:**
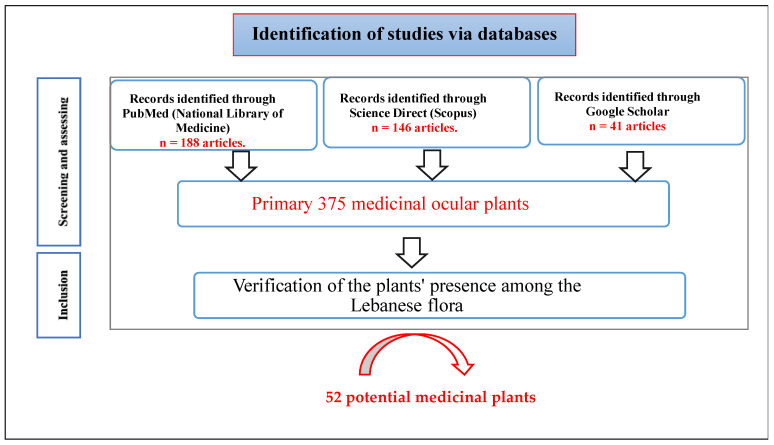
Flow chart of the study design.

Population (P): This study included published articles (original research) related to medicinal plants used to treat ophthalmic disorders covering the period from January 2000 to October 2024 in English. Articles from PubMed (National Library of Medicine) (188 articles), Science Direct Scopus (146 articles), Google Scholar (41 articles), and published books were used to create a primary list of ophthalmic plants, as well as World Flora Online (http://www.worldfloraonline.org, accessed on 10 October 2024) and Plants of the World Online (https://powo.science.kew.org/, accessed on 10 October 2024). The primary list contained the names of nearly 375 medicinal plants. These plants were then checked for their presence in Lebanese flora using the following resources: http://www.lebanon-flora.org/ accessed on 10 October 2024, and the atlas *Nouvelle Flore du Liban et de la Syrie* documenting the biodiversity in these regions [23]. Figure 2 illustrates the different steps involved in the study design.

Inclusion criteria: All original research articles on medicinal plants used to treat different ocular disorders in English and related to plants growing in Lebanon.

Exclusion criteria: All data published outside the timeframe of the study period, studies published in languages other than English, or works on plants not native to the Lebanese flora.

The final dataset, consisting of 52 different species, was compiled in an Excel worksheet (Table 1) and arranged alphabetically. The chemical structures were drawn using ChemDraw Professional 15 software.

## 4. Findings and Discussion

**Figure 3:**
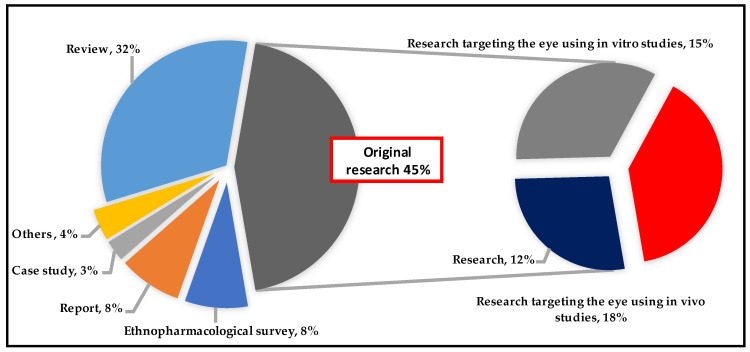
Percentages of article types used in the study.

The compiled papers are from a variety of sources: reviews account for 32%, ethnopharmacological surveys for 8%, reports for 8%, and case studies for 3%. Notably, 45% are classified as original research publications (Figure 3); 18% of these articles focused on the eye in vivo, while 15% of the studies were in vitro. In contrast, 12% of the research did not involve either in vitro or in vivo trials.

The names of the medicinal plants found in the Lebanese flora are listed in Table 1, along with their Latin names, regional names, plant parts used, preparation forms, ocular uses, and related sites of action. The plant names were verified using http://www.theplantlist.org, accessed on 10 October 2024. The various plant characteristics were recorded exactly as provided by the reference sources, without any commentary, to prevent misunderstandings or incorrect interpretations. These species are listed in the order of their appearance in the table: *Althaea officinalis*, *Alhagi maurorum*, *Allium sativum*, *Anagallis arvensis*, *Arbutus unedo*, *Bidens pilosa*, *Borago officinalis*, *Capparis spinose*, *Centaurea cyanus*, *Chenopodium opulifolium*, *Cichorioum intybus*, *Citrullus colocynthis*, *Crepis robertioides*, *Crepis libanotica*, *Cyperus rotundus*, *Datura stramonium*, *Daucus carota*, *Euphrasia officinalis*, *Ficus carica*, *Foeniculum vulgare*, *Fumaria officinalis*, *Hyoscyamus niger*, *Iris germanica*, *Juniperus excelsa*, *Linum usitatissimum*, *Ocimum basilicum*, *Olea europaea*, *Origanum syriacum*, *Origanum laevigatum*, *Malvae sylvestris*, *Matricaria chamomilla*, *Marrubium vulgare*, *Melissa officinalis*, *Mentha longifolia*, *Mentha spicata*, *Nerium oleander*, *Plantago lanceolate*, *Portulaca oleracea*, *Rosa damascena*, *Rosa centifolia*, *Rosemarinus officinalis*, *Salvia sclarea*, *Salvia Libanotica fruticose*, *Salvia officinalis*, *Silybum marianum*, *Solanum dulcamara*, *Solanum villosum*, *Thymus vulgaris*, *Urginea maritima*, *Xanthium strumarium*, *Ziziphus jujube*, and *Ziziphus spina-christi*.

### 4.1. Family Classification

**Figure 4:**
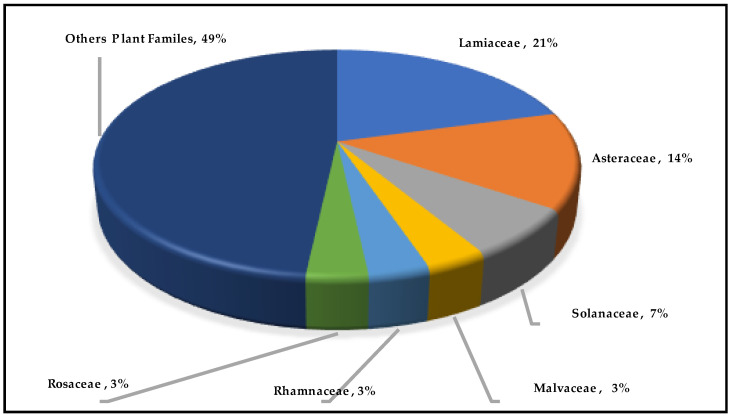
Percentage distribution of cited ocular medicinal plants.

The effectiveness of 52 species from 28 families, including two indigenous ones (*Crepis libanotica* and *Salvia libanotica*), was documented in this study. The majority of species are from the Lamiaceae family (21%), followed by the Asteraceae (14%) and Solanaceae (7%), while Malvaceae, Rhamnaceae, and Rosaceae are present (3% each). Other families accounted for 49% of the total (Figure 4).

Table 1 shows that most of the cited Lebanese medicinal plants have multiple ocular properties. Additionally, many plant families exhibit similar treatment characteristics. Interestingly, the etiology of most ocular diseases involves free radical-mediated oxidative damage, hypoxia, reduced blood supply to ocular tissues, and, in certain conditions, angiogenesis [93]. Phytochemicals show antioxidant, anti-angiogenic, and/or anti-inflammatory activities, and they can also reduce fluid retention and strengthen capillary walls [94]. Therefore, the prevention or treatment of eye disorders may benefit from the selection of these phytochemicals.

To minimize high heterogeneity and traditional publication bias, Table 2 lists the various phytoconstituents found in the listed Lebanese medicinal plants (from research articles only) along with their corresponding mechanisms of action.

Species of the Lamiaceae family have traditionally been used for their curative and preventive properties. Their value stems from the synthesis of a wide range of secondary metabolites with antibacterial, antioxidant, anti-inflammatory, antimicrobial, antiviral, and anticancer properties. The main classes of phenolic compounds identified are phenolic acids, mainly caffeic and rosmarinic acids, and flavonoids. These antioxidant defenses contribute to eye health and maintenance [118]. In this context, the essential oils of *Rosemarinus officinalis*, *Salvia sclarea*, and *Thymus vulgaris,* which contain monoterpene, diterpene, and sesquiterpene hydrocarbons, azulene, alcohols, aldehydes, and ketones, have been cited for their antibacterial properties (Table 2) [80,81,82]. Keratitis caused by the fungus Fusarium can be treated with *Salvia sclarea*, while amoebic keratitis can be treated with ethanol extracts of *Origanum syriacum* and *Origanum laevigatum* [65]. Other beneficial features of this family include its ability to reduce ocular inflammation, as demonstrated by *Marrubium vulgare* [68], *Salvia libanotica fruticose* [83], and *Salvia officinalis* [84].

Long-term exposure to UV, visible ionizing radiation, and environmental toxins cause oxidative damage in ocular tissues, leading to pathological consequences in the ageing eye. While eye tissues, like the tear film, have strong antioxidant defenses against free radicals, the trabecular meshwork lacks these defenses [111]. The inclusion of saturated fatty acids (palmitic and stearic acids) and unsaturated fatty acids (α-linolenic, linoleic, and oleic acids) in *Ocimum basilicum* has beneficial effects. Moreover, polyunsaturated fatty acids can reduce intraocular pressure due to their anti-inflammatory and antioxidant properties. The contributions of flavonoids (quercetin, rhamnocitrin, and luteolin), phenolic acids (rosmarinic and caffeic), and volatile compounds (geranial, neral, citronellal, and geraniol) explain why *Melissa officinalis* shows potential in preventing age-related macular degeneration [27,28]. The antimicrobial activity of *Thymus vulgaris* is attributed to compounds such as α-thujene, α-pinene, and camphene. The essential oil of *T. vulgaris* can inhibit the growth of microscopic filamentous fungi from the genus *Penicillium* [116,117]. In addition, polyphenolic extracts from Spearmint (*Mentha spicata*) may provide nutritional support for neuronal tissues, potentially complementing hypotensive treatments for glaucoma and other ocular conditions by disrupting antioxidant, anti-inflammatory, and neuroprotective mechanisms [72].

The pharmacological effects of Asteraceae plants can be attributed to their wide range of phytochemical compounds, including polyphenols, phenolic acids, flavonoids, acetylenes, and triterpenes. The Asteraceae family exhibits potent antioxidant, anti-inflammatory, and antibacterial properties [119]. Notable species in this category include *Bidens pilosa*, *Centaurea cyanus*, *Cichorioum intybus*, and *Matricaria chamomilla*, which can be used to alleviate eye inflammation. *Crepis robertioides* and *Crepis libanotica* have been used to treat eye infections, while *Centaurea cyanus* is effective specifically in eyelid and conjunctival inflammation such as blepharitis and conjunctivitis. However, the antioxidant properties of silibinin, a flavonolignan extracted from Silybum marianum, make it useful in the treatment of age-related macular degeneration [86,115]. Many eye conditions are treated with *Matricaria chamomilla* [30,31]. Additionally, *Xanthium strumarium* has the potential to enhance vision [90].

The abundance of phytochemicals in the family Solanaceae highlights their potential as healing plants. This family, also known as the nightshade family, includes plants that contain lethal alkaloids [120]. In addition to being an insecticide used to remove Lucilia sericata, *Hyoscyamus niger* can cause red eyes or itching [57]. Joo, 2023, highlighted the dual role of alkaloids as both therapeutic agents and potential toxins [121]. On the other hand, *Solanum dulcamara* and *Solanum villosum* can be used to treat eye inflammation and sore eyes, respectively.

Species of the Malvaceae family possess a wide variety of chemical constituents, such as polysaccharides, coumarins, flavonoids, polyphenols, vitamins, terpenes, and tannins, are found in different plant organs, particularly in the leaves and flowers. These compounds are linked to their biological activity [122]. *Althaea officinalis* and *Malvae sylvestris* are notably known for their ability to moisturize the eyes and prevent dry-eye disease. This effect is attributed to the presence of mucilaginous molecules, which may enhance the lubricating effect [35,36].

The health benefits of the Rosaceae family are linked to its secondary metabolites, with enriched extracts finding applications in pharmacology. Its main constituents include flavonoids, triterpenes, tannins, polysaccharides, phenolic acids, fatty acids, organic acids, carotenoids, and vitamins [123]. The flower extracts of *Rosa damascena* and *Rosa centifolia* are used for eye washing and treating eye inflammation. Terpenoids and saponins, which are abundant in the genus Ziziphus, are responsible for many of its health benefits [124]. Rhamnaceae plants *Ziziphus jujube* and *Ziziphus spina-christi* have also been utilized to treat eye inflammation.

### 4.2. Plant Parts

**Figure 5:**
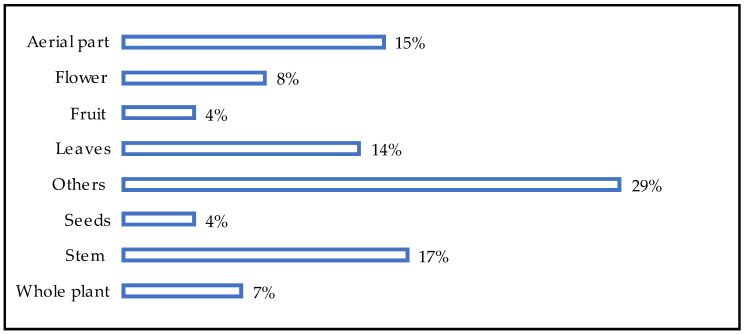
Percentage distribution of plant parts used to treat ocular diseases.

Most plant components are applied topically to the affected area of the eye and utilized externally, although some are also used internally. The stem was prevalent in the remedies that we found (17%), followed by aerial parts (15%), leaves (4%), and flowers (8%) (Figure 5).

### 4.3. Ocular Preparation and Administration

The 52 medicinal plants cited in this study were prepared and administered using a variety of techniques, as shown in Table 1. These methods are accurately reported as cited in the references. Infusion, decoction, or maceration in water are the basic methods used to obtain aqueous plant extracts. A decoction is typically used for the harder or woodier parts of the plant; it involves placing the plant in cold water, roughly divided, and boiling the mixture for at least 15 min over a moderate flame [125]. *Anagallis arvensis* leaf juice can be used to improve eyesight, while an infusion of *Borago officinalis* leaves can treat conjunctivitis. Both the flower of *Fumaria officinalis* and the rhizome decoction of *Cyperus rotundus* can be used as alternative remedies for conjunctivitis. Eyebright, also known as *Euphrasia officinalis*, has historically been used in folk medicine mostly to treat eye conditions.

Additionally, solvents can penetrate plant cell walls to release intracellular compounds, thereby increasing the yield of the extracts. Studies have shown that using alcohol-based solvents, especially in aqueous mixtures (e.g., 80% methanol or ethanol), can improve the extraction of phenolic content and antioxidant activities [126]. It has been suggested that the methanolic extracts of *Citrullus colocynthis*, *Allium sativum*, and *Iris germanica* could delay the development of cataracts, reduce intraocular pressure, and treat conjunctivitis. Additionally, methanolic extracts of *Origanum basilicum* and *Origanum syriacum* have been linked to the treatment of amoebic keratitis.

Essential oils (EOs) are complex mixtures of secondary metabolites with specific chemical compositions that vary according to the plant’s characteristics. In the East, aromatic substances are often considered more than just perfumes and are used for therapeutic purposes [81]. The essential oils of *Thymus vulgaris*, *Salvia sclarea*, *Rosemarinus officinalis*, and *Salvia officinalis* have also been cited for their numerous ocular qualities, particularly their antibacterial effects.

Some formulations were cited in specific forms, such as lotions (*Plantago lanceolate* and *Fumaria officinalis*) and ointments (*Chenopodium opulifolium* and *Olea europaea*). When blended with sugar, *Alhagi maurorum* flower powder is used to cleanse the eye and improve vision. *Linum usitatissimum* oil capsules have been used to treat patients with dry-eye and Sjögren’s syndrome. The simple edible plants mentioned include *Arbutus unedo*, *Daucus carota*, *Foeniculum vulgare*, and *Portulaca oleracea*.

### 4.4. Disease Treatment Classification

Recent preclinical studies have explored the use of re-emerging herbal compounds for prophylaxis and management in anterior- and posterior-segment eye diseases [127]. A significant percentage (68%) of the cited Lebanese medicinal plants could be used to target the anterior portion of the eye. The most frequently recommended treatments were for eye infections or inflammation, leading to symptoms such as pain, redness, discharge, excessive moisture, and light sensitivity. These characteristics are commonly observed in species belonging to the Lamiaceae and Asteraceae families, as previously mentioned. Certain plants, such as *Bidens pilosa*, *Linum usitatissimum*, *Malvae sylvestris, Melissa officinalis,* and *Olea europaea*, have been found to play a role in stabilizing the tear film, thus reducing dry-eye symptoms. *Daucus carota*, rich in carotenoids such as beta-carotene, lutein, and zeaxanthin, helps protect the eye from oxidative stress, apoptosis, mitochondrial dysfunction, and inflammation. Additionally, *Matricaria chamomilla* may offer protection against ultraviolet (UV) radiation or cataract development (Table 2). A lower incidence of age-related eye disorders is associated with a high intake of these carotenoids. These phytomolecules are lipophilic, meaning they can penetrate biological barriers like the blood–retina barrier (BRB). As a result, their ability to reach the retina enables them to have anti-inflammatory and antioxidant effects [128].

However, 5% of the listed medicinal plants, including *Chenopodium opulifolium*, *Juniperus excels,* and *Urginea maritima*, are suggested for use in treating unspecified eye disorders.

**Figure 6:**
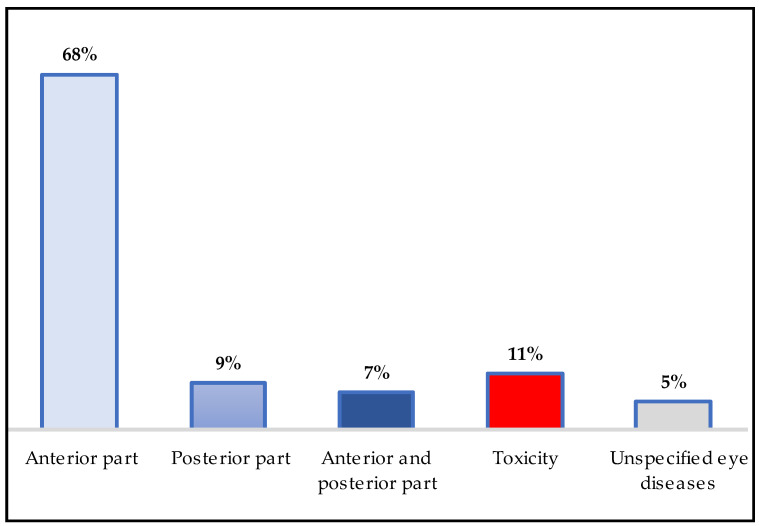
Percentage distribution of sites of action of medicinal plants.

The complex structure and physiology of the eye make it difficult for ophthalmologists to treat posterior-segment ocular diseases such as age-related macular degeneration (AMD) or diabetic retinopathy (DR) [129]. As illustrated in Figure 6, only 9% of the cited medicinal plants are effective for conditions such as glaucoma or AMD. The specialized nature of the eye includes several static and moving obstacles that prevent drugs from reaching their intended target sites of action. Therefore, new carrier systems for herbal phytoconstituents should be developed to overcome the poor permeability and absorption limitations at the desired site of action [127]. Research indicates that *Arbutus unedo* can delay or prevent cataracts due to the presence of zeaxanthin [130,131]. *Borago officinalis* contains gamma-linolenic acid, a vasodilator that improves blood flow and may help lower retinal venous pressure [34]. *Melissa officinalis* (Lamiaceae) is used for age-related macular degeneration (AMD) due to its volatile compounds (geranial, citronellal, and geraniol), phenolic acids (rosmarinic and caffeic acid), and flavonoids (quercetin, rhamnocitrin, and luteolin). These phytochemicals can reduce apoptosis and oxidative damage while exhibiting potent antioxidant properties, acting as radical scavengers [69,113]. Additionally, phenolic compounds derived from *Mentha spicata* have been linked to improvements in neurotrophin levels, along with reductions in oxidative stress and inflammation markers, making them beneficial in treating glaucoma [72]. Finally, *Silybum marianum* is effective in treating age-related macular degeneration (AMD), primarily due to its flavonolignans, which possess antioxidant properties [86,115].

Treating posterior-segment ocular illnesses can be challenging; however, drug permeability improves with increased lipophilicity and decreases with lower molecular weight and/or reduced protein binding [129]. Table 3 lists the main phytochemical components along with their solubility, molecular weight, and functional groups. Plants that target both the anterior and posterior parts simultaneously include *Allium sativum* [132], *Foeniculum vulgare* [55], *Rosa damascene* [133], and *Rosemarinus officinalis* [134]. For example, solubility affects their capacity to cross ocular barriers, and functional groups determine how they interact with biological targets. Most of the listed molecules are slightly soluble in water but exhibit higher solubility in organic solvents. Additionally, their molecular weight ranges from 148 to 360 Da. Understanding these characteristics aids in the development of efficient ocular drug delivery systems [135].

### 4.5. Toxicology of Some Cited Medicinal Plants

The common belief that herbal remedies have no side effects has led to the widespread use of traditional eye medications. However, this dangerous misuse can result in ocular morbidity due to close contact with the eyes. Herbal eye “medicines” are believed to cause 8-10% of corneal blindness in Africa [137]. The negative effects include a worsening of the initial condition and an increased risk of infections, which, in extreme cases, can result in total eye injury. Since 11% of the described plants show signs of toxicity, caution should be exercised when handling plants that can harm the eyes, such as *Datura stramonium*, *Ficus carica*, *Hyoscyamus niger*, *Nerium oleander*, and *Urginea maritima*. The primary hazardous phytochemicals are listed in Table 4. Alkaloids such as atropine, scopolamine, and hyoscyamine are responsible for blocking the muscarinic acetylcholine receptors in the iris sphincter muscle, preventing contracting [138]. Some documented adverse outcomes include photophobia, mydriasis, blurred vision, and eye pain.

Additionally, the branches, leaves, and fruit skin of the fig tree (*Ficus carica*) release a milky sap or latex that contains proteolytic enzymes and furocoumarins. These compounds are known to be photoirritants [51].

## 5. Conclusions

Thousands of medicinal plant species have significant economic, social, and ecological value and are fundamental to human well-being. The 52 Lebanese medicinal plants listed in this study could play a crucial role in the development of new, affordable, and effective ocular drugs. This study was the first on this topic and established a foundation for further phytochemical and pharmacological exploration.

However, significant research gaps were identified, particularly in the studies focusing on ocular medicinal plants and eye diseases. Notably, 33% of the studies involved in vivo or in vitro trials, indicating the need for more thorough investigations. While phytochemical products have shown promise as potential therapies, many remain untested or inadequately monitored. Additionally, the efficacy of herbal treatments can be highly variable due to differences in their sources, quality, combinations, and preparation methods. This lack of standardization may lead to potential adverse reactions. The safety of herbal medications remains a major concern, and regulatory bodies must ensure that all herbal medicines are safe and of acceptable quality [1].

Endemic plants, such as *Crepis libanotica* and *Salvia libanotica*, should be screened through standardized pharmacological and clinical procedures to assess their potential activities [7]. Consequently, research into the phytochemical profiles of these species, particularly those that are currently underexplored, can aid in discovering bioactive compounds with positive effects on ocular health while also uncovering the potential uses and economic importance of these plants.

## Figures and Tables

**Table 1:** Ocular medicinal plants found in the Lebanese flora.

	Scientific Name	Local Name	Family	Part Used	Preparation	Ocular Treatments	Category	Type of Article	Reference
1	*Althaea officinalis*	El Khaimah	Malvaceae	Flower	NA	Eye inflammation	Anterior part	Traditional medicine	[24]
				Leaves		Puffy and swollen eyelids		Review	[25]
						Conjunctivitis			
						Eye discharge			
						Hordeolum			
2	*Alhagi maurorum*	Chawk El Jamal	Papilionaceae	Flower	Ground flowers powdered with sugar	Cleaning the eye	Anterior part	Ethno-medicine	[26]
						Improving eyesight			
3	*Allium sativum*	Sin El Thoum	Liliaceae	Bulb	Methanolic extract	Preventing/delaying cataracts	Anterior and posterior part	Research on the eye using in vivo study	[27]
					Tablet	Diabetic retinopathy		Research on the eye using in vivo study	[28]
						Lowering IOP			
						Improving visual acuity			
						Adjuvant treatment in diabetic macular edema			
4	*Anagallis arvensis*	Ain El Jamal	Primulaceae	NA	Plant juice	Ophthalmia Kertistis	Anterior part	Literature survey and fieldwork	[29]
						Improving eyesight			
5	*Arbutus unedo*	Quotlob	Ericaceae	Fruit	Edible	Preventing/delaying cataracts	Posterior part	Review (Zeaxanthin occurrence)	[30]
						Age-related macular degeneration			
6	*Bidens pilosa*	Hsseiki	Asteraceae	Plant juice	Plant juice	Eye irritation	Anterior part	Review	[31]
						Conjunctivitis			
				Leaves	Infusion	Improving aqueous tear quantity	Anterior part	Research on the eye using in vivo study	[32]
						Maintaining tear film stability			
						Inhibiting the inflammation of the lacrimal gland			
						Maintaining tear film stability			
7	*Borago officinalis*	Lissan El Thawr	Boraginaceae	Leaves	Infusion	Conjunctivitis	Anterior part	Ethnobotanical survey	[33]
				Flower					
				Flower	Infusion	Reduce retinal venous pressure	Posterior part	Research on the eye using in vivo study	[34]
8	*Capparis spinosa*	El Quoubar	Capparidaceae	Leaves	Infusion	Eye infection	Anterior part	Review	[35]
				Bud	Orally with a glass of water				
9	*Centaurea cyanus*	El Quantarioun	Asteraceae	Flower	Eyewash with cornflower	Eye inflammation	Anterior part	Review	[36]
					Infused blossoms	Conjunctivitis			
						Blepharitis			
						Relieve strained tired and puffy eyes			
10	*Chenopodium opulifolium*	Sarmouc	Chenopodiaceae	Leaves	Ointment	Eye diseases	Unspecified eye diseases	Research	[37]
11	*Cichorioum intybus*	El Hindbeh	Asteraceae	Flower	Infusion	Eye inflammation	Anterior part	Review	[38]
						Periorbital puffiness			
						Symptoms of eye tiredness			
				Leaves	External application	Eye infection		Review	[39]
				Root					
				Juice					
12	*Citrullus colocynthis*	El Hanzal	Cucurbitaceae	Fruit	Methanolic extracts	Eye redness	Anterior part	Research on the eye using in vitro study	[40]
						Preventing/delaying cataracts			
13	*Crepis robertioides*	Saraghat Robertieih	Asteraceae	Flowering part	Infusion	Eye infection	Anterior part	Ethnopharmacological survey	[41]
14	*Crepis libanotica*	Saraghat Lebnen	Asteraceae	Flowering part	Infusion	Eye infection	Anterior part	Ethnopharmacological survey	[41]
15	*Cyperus rotundus*	El Saad	Cyperaceae	Rhizome	Decoction	Eye disease		Review	[14]
						Ocular discharges	Anterior part	Review	[42]
16	*Datura stramonium*	El Khawkhara	Solanaceae	Seeds	Extraction	Mydriasis	Toxicity	Research	[43]
				Leaves	Smoking	Photophobia			
17	*Daucus carota*	El Jazar	Umbelliferae	Root	Edible	Protecting against chronic eye defects and vision loss	Anterior part	Review	[44,45]
						Protection against UVB Improving eyesight			
				Seeds	Extraction	Lower internal ocular pressure (IOP)		Research on the eye using in vivo study	[46]
18	*Euphrasia officinalis*	Arkoun Toubi	Orobanchaceae	Whole plant	Tincture Extraction with ethanol Herbal tea	Conjunctivitis	Anterior part	Assessment report	[47]
						Blepharitis			
						Eye fatigue			
						Ocular inflammation			
						Styes			
						Ocular allergies			
				Commercial eye drops		Protecting corneal epithelial cells from UVB exposure		Research on the eye using in vitro study	[48]
					Methanol and ethanol extract	Anti-inflammatory Reducing pro-inflammatory cytokine expression		Research on the eye using in vitro study	[49]
19	*Ficus carica*	El Tin	Moraceae	Stem	Sap of the plant	Used in eye irritation	**Toxicity**	Case study	[50,51]
				Fruit	Edible fruit	Improving eyesight	Anterior part	Review	[52]
					Powder of dry fruits and sugar taken orally with water twice a day				
20	*Foeniculum vulgare*	El Choumar	Apiaceae	Seeds	Water seed extract	Reducing intraocular pressure (IOP)	Anterior and posterior part	Research on the eye using in vivo study	[53]
						Anti-glaucoma			
						Protective and therapeutic effects against induced cataracts		Research on the eye using in vivo study	[54]
					Raw or with a sweetener	Improving eyesight		Review	[55]
21	*Fumaria officinalis*	Bakleht El Malak	Papaveraceae	Stem	Eye lotion	Red eye	**Toxicity**	Book chapter	[56]
				Flower	NA	Conjunctivitis			
						Heaviness in the eyes,			
						Stinging pain in the eyes			
						Swelling and puffiness of the eyes			
						Photophobia and tired eyes			
22	*Hyoscyamus niger*	El Binij El Aswad	Solanaceae	Seeds	Extraction	Insecticidal activity (*Lucilia sericata*)	Anterior part	Research	[57]
						Red eye			
						Itching in the eye			
						Mydriasis	**Toxicity**	Review	[58]
						Blurred vision			
						Photophobia			
23	*Iris germanica*	El Sawsan	Iridaceae	Leaves	Methanol extract	Conjunctivitis	Anterior part	Research on the eye using in vitro study	[59]
						Eye infection			
24	*Juniperus excelsa*	El Charbin	Cupressaceae	Seeds	Seed extract	Eye diseases	Unspecified eye diseases	Review	[14]
25	*Linum usitatissimum*	El Kittein	Linaceae	Seeds	Seed mucilage	Removing foreign material from the eye	Anterior part	Assessment report	[60]
						Eye irritation			
					Oral flaxseed oil capsules	Dry-eye Sjögren’s syndrome patients		Research on the eye using in vivo study	[61]
26	*Ocimum basilicum*	El Rihan	Lamiaceae	Seeds	Aqueous extract	Lower internal ocular pressure (IOP)	Anterior part	Research on the eye using in vivo study	[62]
27		El Zaytoun	Oleaceae	Leaves	Infusions used as ointment	Eye infections	Anterior part	Research	[63]
						Management of dry-eye syndrome		Review	[64]
28	*Origanum syriacum*	Zaatar souri	Lamiaceae	Arial parts	Methanol extract	Treatment for amoebic keratitis	Anterior part	Research	[65]
29	*Origanum laevigatum*	Mardakouch	Lamiaceae	Arial parts	Methanol extract	Treatment for amoebic keratitis	Anterior part	Research	[65]
30	*Malvae sylvestris*	Khibeizi	Malvaceae	Whole plant	Mucilaginous extract	Dry-eye disease	Anterior part	Research on the eye using in vivo study	[66]
31	*Matricaria chamomilla*		Asteraceae	Flower	Infusion	Protecting against UVB exposure	Anterior part	Research on commercial eye drops (Dacriovis™)	[48]
				Leaves	Decoction	Eye irritation or eye infection		Review	[67]
						Eyewash			
						Eye care			
						Swollen eyes			
						Tired eyes			
						Ameliorating wound healing			
32	*Marrubium vulgare*	Kourrat Jabali	Lamiaceae	Leaves	Extraction	Eye inflammation	Anterior part	Review	[68]
						Sore eyes			
					Leaf juice with honey	Night blindness			
						Clean eyesight			
33	*Melissa officinalis*	El Mleissi	Lamiaceae	Leaves	Aqueous ethanol extraction	Dry age-related macular degeneration (AMD)	Posterior part	Research on the eye using in vitro study	[69]
						Exudative AMD		Research	[70]
34	*Mentha longifolia*	Alnaenae al Tawil	Lamiaceae	Aerial part Leaves	Infusion	Eye diseases	Anterior part	Ethnopharmacological survey	[71]
35	*Mentha spicata*	Alnaenae al Akhdar	Lamiaceae	Extract	Marketed as Neumentix	Nutritional support in a rat model of hypertensive glaucoma	Posterior part	Research on the eye using in vivo study	[72]
36	*Nerium oleander*	El Delfi	Apocynaceae	Leaves	Sap of the plant	Eye inflammation	**Toxicity**	Case study	[73]
						Light sensitivity			
						Keratitis and uveitis			
						Corneal edema			
37	*Plantago lanceolata*	Lissan El Hamal	Plantaginaceae	Whole plant	Lotion	Eye illness wound repair	Anterior part	Report	[74]
				Leaves	Ointment Eye drops	Eye irritation		Review	[75]
						Eye choroid diseases			
						Day blindness			
						Conjunctivitis			
						Eyes sores			
38	*Portulaca oleracea*	Bakleh Barrieh	Portulacaceae	Leaves	Edible plant	Inflammation of the eyes	Anterior part	Review	[76]
				Seeds	Decoction				
39	*Rosa damascena*	Ward Dimachqy	Rosaceae	Flower	Rose water	Eye wash	Anterior and posterior part	Review	[77]
						Eye inflammation		Research on the eye using in vivo study on eye drop preparation (Ophthacare^®^)	[78]
						Degenerative ophthalmic disorders			
40	*Rosa centifolia*	Ward Outri	Rosaceae	Flower	Infusion	Eye wash	Anterior part	Assessment report	[79]
						Eye inflammation			
41	*Rosemarinus officinalis*	Eklil El Jabal	Lamiaceae	Whole plant	Essential oil	Prevention of retinal light damage	Anterior and posterior part	Research on the eye using in vivo study	[80]
						Antibacterial activity		Research on the eye using in vitro study	[81]
						Antimicrobial activity			
42	*Salvia sclarea*	Qasein	Lamiaceae	Leaves	Essential oil	Antimicrobial activity	Anterior part	Research on the eye using in vitro study	[81]
						Antifungal effect (fusarium keratitis)		Research on the eye using in vitro study	[82]
43	*Salvia Libanotica fruticosa*	Mariamia Loubnenieh	Lamiaceae	Leaves	Infusion	Eye inflammation	Anterior part	Short communication	[83]
44	*Salvia officinalis*	Qasein Toubi	Lamiaceae	Arial parts	Essential oil	Eye disease	Anterior part	Research	[84]
				Arial parts	Vapor inhalation	Ophthalmic anti-inflammatory		Ethnopharmacological and chemical characterization	[85]
				Leaves	NA				
45	*Silybum marianum*	Kharfeich	Asteraceae	Whole plant	Isolation of Silibinin	Age-related macular degeneration	Posterior part	Research on the eye using in vitro study	[86]
						Neovascular AMD			
46	*Solanum dulcamara*	Bathenjein Aswad	Solanaceae	Stem	Lipstick	Mydriasis	**Toxicity**	Case report	[87]
47	*Solanum villosum*	Bathenjein Ahmar	Solanaceae	Whole plant	Edible	Sore eyes	Anterior part	Review	[88]
48	*Thymus vulgaris*	Zaatar Bari	Lamiaceae	Whole plant	Essential oil extraction Steam distillation	Antimicrobial activity	Anterior part	Research on the eye using in vitro study	[81]
49	*Urginea maritima*	Basal Bari	Asparagaceae	Bulb	Fresh	Eye illness	Unspecified eye diseases	Report	[89]
50	*Xanthium strumarium*	El Lizeiq	Asteraceae	Leaves	NA	Eye diseases	Anterior part	Review	[90]
				Seeds		Improving eyesight			
51	*Ziziphus jujuba*	El Inab	Rhamnaceae	Seeds	Extraction	Eye inflammation	Anterior part	Review	[91]
52	*Ziziphus spina-christi*	El Sidir	Rhamnaceae	Leaves	Extraction	Eye inflammation	Anterior part	Review	[92]

NA (not available).

**Table 2:** Phytoconstituents and mechanisms of action of the listed medicinal plants (from research articles focused on the eyes).

Plant Source	Family	Disease or Target	Phytochemical(s)	Structure	Mechanism of Action	Reference
*Allium sativum*	Liliaceae	Cataracts	Allicin	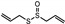	Antioxidant activity prevents protein modifications in cataractous lenses	[95]
			S-allyl cysteine	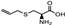	Antioxidant and anti-inflammatory properties Protection against neurodegenerative diseases	[96]
		Diabetic retinopathy	S-allyl cysteine		An antidiabetic mechanism inhibits angiogenesis by downregulating vascular endothelial growth factor (VEGF) expression	[97]
			Allicin		Allicin delays the progression of diabetic nephropathy through antioxidant and anti-inflammatory mechanisms	[98]
			Diallyl disulfide (DADS)	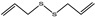	Anti-inflammatory and antioxidant mechanisms	[99]
			Flavonoids	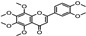	Anti-oxidative, anti-inflammatory, and anti-apoptotic mechanisms Inducing heme oxygenase-1 expression	[100]
*Borago officinalis*	Boraginaceae	Reducing retinal venous pressure	Gamma-linolenic acid	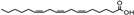	Intermediate of PGE1, an endogenous vasodilator that enhances blood flow	[34]
			Phenolic Compounds	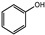 Phenol	Improving blood flow	
*Citrullus colocynthis*	Cucurbitaceae	Preventing/delaying cataracts	Flavonoids (Quercetin)	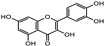	Oxidative stress leads to protein aggregation in the lens, which causes cataracts Quercetin’s antioxidant activity can help protect lens proteins from oxidation	[101,102]
			Phenolic compounds	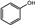 Phenol	Antioxidant and free radical scavenging	[103]
*Daucus carota*	Umbelliferae	Protecting against chronic defects and vision loss	Carotenoids (beta-carotene, lutein, and zeaxanthin)	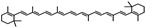 Beta-carotene 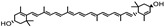 Lutein	Protecting the eye from oxidative stress, apoptosis, mitochondrial dysfunction, and inflammation	[104]
*Euphrasia officinalis*	Orobanchaceae	Protecting corneal epithelial cells against UVB exposure Anti-inflammatory effects	Phenolic compounds gentisic, caftaric, vanillic and rosmarinic acid, hyperoside (quercetin-3-O-galactoside), and quercitrin (quercetin 3-O-rhamnoside)	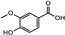 Vanillic acid 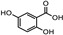 Gentisic acid 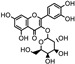 Quercetin-3-O-galactoside 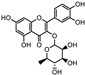 Quercetin 3-O-rhamnoside	Antioxidant, antimicrobial and antiproliferative activities	[105]
*Foeniculum vulgare*	Apiaceae	Reducing intraocular pressure (IOP)	NA		Anticholinesterase activity, which may help lower IOP	[53]
		Protective and therapeutic effects against induced cataracts	Anethole Fenchone Flavonoids	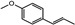 Anethole 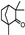 Fenchone	Inhibiting oxidative stress and preventing lens opacity Antioxidant and anti-inflammatory activities	[54,106,107]
*Iris germanica*	Iridacea	Eye infection	Phenolic compounds: Protocatechuic acid, Catechin, p-Hydroxy benzoic acid, and caffeic and Ferulic acid	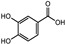 Protocatechuic acid 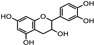 Catechin 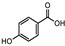 p-HydroxyBenzoicacid 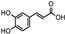 Caffeic acid 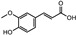 Ferulic acid	Antimicrobial, antioxidant, and antimutagenic activities	[108]
*Linum usitatissimum*	Linaceae	Dry-eye Sjögren’s syndrome patients	Essential fatty acids (EFAs), particularly omega-3	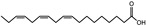 α-linolenic acid	Improving tear production and reducing inflammation	[109,110]
*Ocimum basilicum*	Lamiaceae	Lower internal ocular pressure (IOP)	Unsaturated fatty acids including α-linolenic, linoleic, and oleic acids Saturated fatty acids (palmitic and stearic acid)	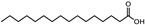 Palmitic acid 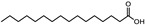 Stearic acid 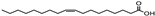 Oleic acid 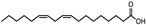 Linoleic acid 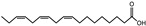 α-linolenic acid	Anti-inflammatory effects Reduction in aqueous humor production	[111]
*Malvae sylvestris*	Malvaceae	Dry-eye disease	Plant mucus		Mucilaginous substances have the potential to support the lubricating effect	[66,112]
			Phenols, flavonoids, carotenoids, unsaturated fatty acids	See above structures	Antioxidants	
*Melissa officinalis*	Lamiaceae	Age-related macular degeneration (AMD)	Volatile compounds (geranial, neral, citronellal, and geraniol) Phenolic acids (rosmarinic and caffeic acid) Flavonoids (quercetin, rhamnocitrin, and luteolin)	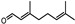 Geranial 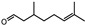 Citronellal 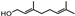 Geraniol	Reducing apoptosis and oxidative damage Potent antioxidant properties and ability to act as a radical scavenger	[69,113]
*Mentha spicata*	Lamiaceae	Glaucoma	Phenolic compounds		Improving levels of neurotrophins, along with reducing oxidative stress and inflammation markers	[72]
*Rosemarinus officinalis*	Lamiaceae	Prevention of retinal light damage Antibacterial activity	Essential oil components including monoterpene, diterpene, and sesquiterpene hydrocarbons, azulene, alcohols, aldehydes, and ketones	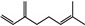 Monoterpene myrcene 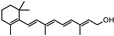 Diterpene retiol 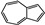 Azulene	Antimicrobial activity of EO	[80,81]
			Carnosol, carnosic, rosmanol, rosmarinic and ursolic acid	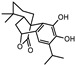 Carnosol 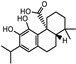 Carnosic acid	Antioxidants	
*Salvia sclarea*	Lamiaceae	Antimicrobial and antifungal effects	Essential oil components include monoterpene, diterpene, and sesquiterpene hydrocarbons and azulene,	See above structures	Antimicrobial activity of EO	[81,114]
*Silybum marianum*	Asteraceae	Age-related macular degeneration	Flavonolignans, silymarin (silybin, isosilybin, silychristin, dihydrosilybin, and silydrianin)	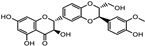 silybin 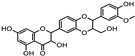 Isosilybin	Antioxidant properties	[86,115]
*Thymus vulgaris*	Lamiaceae	Antimicrobial activity	Essential oil components including monoterpene, diterpene, and sesquiterpene hydrocarbons, azulene, alcohols, aldehydes, ketones, α-thujene, α-pinene, and camphene	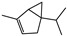 α-thujene 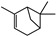 α-pinene 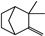 Camphene	Antimicrobial activity of EO	[81,116,117]

**Table 3:** Main phytochemical components of some listed Lebanese medicinal plants acting on the anterior and posterior eye segment simultaneously. Properties were retrieved from PubChem [136].

Medicinal Plant	Compound	Solubility	Molecular Weight (Daltons)	Functional Groups	Pubchem CID
*Allium sativum* [132] ** 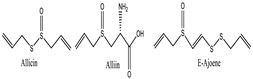 **	Allicin	Soluble in alcohol and organic solvents; sparingly soluble in water	162.19	Thioester, alkene (C=C), eher	CID: 6391
	Allin	Soluble in water and organic solvents	177.21	Sulfide, amine	CID: 6320
	E-Ajoene	Soluble in alcohol and organic solvents, low solubility in water	206.33	Disulfide, alkene (C=C)	CID: 10632
*Foeniculum vulgare* [55] 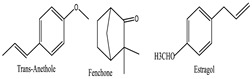	Trans-Anethole	Soluble in alcohol and lipids, sparingly soluble in water	148.21	Ether (-OCH₃), vinyl group (C=C)	CID: 10203
	Fenchone	Soluble in organic solvents, practically insoluble in water	150.22	Ketone (C=O)	CID: 6395
	Estragole	Soluble in organic solvents, low solubility in water	148.21	Ether (-OCH₃), alkene (C=C)	CID: 9887
*Rosa damascene* [133] 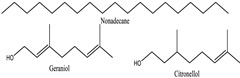	Nonadecane	Insoluble in water but soluble in organic solvents	270.45	Alkane	CID: 6295
	Geraniol	Soluble in alcohol and lipids, low solubility in water	154.25	Alcohol (-OH), alkene (C=C)	CID: 10445
	Citronellol	Soluble in alcohol and oils, low solubility in water	154.25	Alcohol (-OH), alkene (C=C)	CID: 10242
*Rosemarinus officinalis* [134] 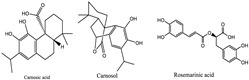	Carnosic Acid	Soluble in organic solvents, insoluble in water	330.46	Carboxylic acid (-COOH)	CID: 5281642
	Carnosol	Soluble in organic solvents, insoluble in water	316.45	Alcohol (-OH), ketone (C=O)	CID: 5281643
	Rosmarinic Acid	Soluble in water and organic solvents	360.36	Ester, carboxylic acid (-COOH)	CID: 442800

**Table 4:** Phytoconstituents and mechanisms of action of toxic medicinal plants.

Plant Source	Disease	Phytochemical(s)	Mechanism of Action	Reference
***Datura stramonium *** 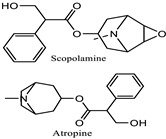	Mydriasis, photophobia	Alkaloids: atropine, scopolamine, and hyoscyamine	Alkaloids work by blocking the muscarinic acetylcholine receptors in the iris sphincter muscle, which results in prolonged pupil dilation	[43,138]
***Ficus carica*** 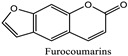	Eye irritation	Furocoumarins: psoralen and bergapten.	Can cause skin and eye irritation Photosensitizing properties, which can lead to photodermatitis	[51]
***Hyoscyamus niger *** 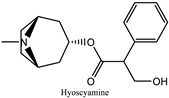	Red eye Mydriasis	Alkaloids, primarily hyoscyamine, atropine, and scopolamine	Anticholinergic activities	[43,138]
***Nerium oleander*** 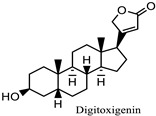	Eye inflammation, Light sensitivity, keratitis, and uveitis Corneal edema	Cardiac glycosides oleandrin, nerin, and digitoxigenin	Can irritate skin and mucous membranes, including the eyes	[73,139]
***Solanum dulcamara*** 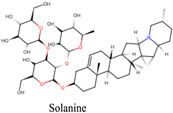	Mydriasis	Tropane alkaloids, such as solanine and other glycoalkaloids	Anticholinergics can cause pupil dilation by blocking the action of acetylcholine	[140]

## Abbreviations

ACS	American Chemical Society
AMD	Age-related macular degeneration
DM	Diabetes mellitus
DR	Diabetic retinopathy
EOs	Essential oil
IOP	Internal ocular pressure
MAPs	Medicinal and aromatic plants
PSED	Posterior-segment eye disease
TFOS DEWS II	Tear Film and Ocular Surface Society Dry Eye Workshop II
VEGF	Vascular endothelial growth factor
TM	Trabecular meshwork
UV	Ultraviolet radiation
